# Intraocular Pressure Following Intravitreal Injection of Triamcinolone Acetonide§

**DOI:** 10.2174/1874364100802010119

**Published:** 2008-06-30

**Authors:** Ejaz A Ansari, N Ali

**Affiliations:** Maidstone and Tunbridge Wells NHS Trust, Department of Ophthalmology, Maidstone Hospital, Hermitage Lane, Maidstone, ME16 9QQ, UK

## Abstract

**Background:**

To investigate the intraocular pressure (IOP) response following intravitreal injection of triamcinolone acetonide.

**Methods:**

This retrospective consecutive non-comparative case series study included 41 patients (52 eyes) (19 male, 22 female, mean age 64.1 ± 13.44; range 22 – 85 years) with progressive exudative ARMD (n = 10 eyes) or diffuse diabetic macular oedema (42 eyes), who received one or more intravitreal injection(s) of 4 mg triamcinolone acetonide.

**Results:**

IOP increased significantly (p<0.001) from 16.08 (±3.28) mm Hg (range 12-26 mm Hg) preoperatively to a mean maximum of 26.1 (±11.79) mmHg (range 15-80 mm Hg) postoperatively (p<0.001). An IOP rise to values higher than 21 mm Hg was observed in 28 (53.8%) eyes. Elevation of IOP occurred 7.5 weeks (±7.07) after the injection. All five patients (11.9%) with a family history of glaucoma developed an IOP rise above the mean maximum level. The post-injection rise of IOP was statistically independent of gender (p=0.37), but the presence of diabetes mellitus demonstrated a marked influence on the rate of a postoperative elevation of IOP (p=0.05).

**Conclusion:**

The IOP response following IVTA was consistent with previous studies. A family history of glaucoma and a history of diabetes mellitus may predispose patients to a greater than average IOP rise following IVTA. Careful IOP assessment for at least 6 months post injection is recommended.

## INTRODUCTION

As the clinical application of intravitreal triamcinolone (IVTA) evolves, so does the interest in its potential side effects and safety. IVTA is effective when used for the treatment of cystoid macular oedema caused by diabetic maculopathy [1], central retinal vein occlusion [2], uveitis, post cataract surgery macular oedema [3]. As IVTA can be considered as a therapeutic approach for a large spectrum of clinical conditions, knowledge of the subsequent IOP response needs to be established, since steroids are associated with IOP increase [4].

Currently there has not been a demonstrable long-term toxic effect of intravitreal triamcinolone injections. However, one recognized and established side effect is the elevation of intraocular pressure (IOP) leading to a secondary chronic open angle glaucoma. A steroid induced glaucoma may occur in up to 52% of eyes after triamcinolone injection [4].

The exact pathophysiology of steroid induced glaucoma is not entirely understood. The elevation in intra-ocular pressure is thought to be due to increased resistance to aqueous outflow. Indisputably however, a rise in IOP following intravitreal steroid injection does occur, and this has been demonstrated by many studies [4-8]. A greater understanding of the dynamics of this elevation, including its timing and extent, is necessary to enable clinicians to detect an IOP rise early and to initiate prompt treatment to prevent permanent damage to the optic nerve head.

Most of the previous studies were from centres in mainland Europe and USA, and we have complemented these with a British study from a large centre in the South-East of England with a predominantly Caucasian population.

## METHOD

The retrospective interventional case series study included 41 patients (52 eyes) (19 men, 22 women; 21 right eyes 31 left eyes) who consecutively received one or more injections of 4 mg intravitreal triamcinolone in topical anaesthesia and had a minimal follow-up of three months. All patients were Caucasian. The injection was given to patients who had reduced visual acuity due to either exudative macular degeneration with subfoveal neovascularization (n = 10 eyes) or diffuse diabetic macular oedema (n = 42 eyes). Eleven patients (11/41) received the intravitreal cortisone injection in both eyes, with a time interval of 1 to 6 months between the injections. Mean age was 64.07 (13.44) years (range 22-85; median 53.5 years). All patients were fully informed about the potential side effects of the therapy and signed an informed consent.

Two patients (0.05%) had primary open angle glaucoma (POAG) before inclusion into this study. Arterial hypertension was present in 10 patients (22.7%). None of the patients in the study had a history of an elevation of IOP during topical or systemic treatment before.

All patients received an intravitreal injection of 4 mg of crystalline triamcinolone acetonide in 0.1 ml Ringer’s solution in the minor operating theatre. The solution was prepared by the hospital’s pharmacy removing the solvent agent. The injection was administered transconjunctivally under topical anesthesia with G. Amethocaine 1% after a paracentesis had been performed to decrease the volume of the globe. Using applanation tonometry, IOP was determined before, and at intervals of 2 - 4 weeks after the injection.

The mean follow up time for the patients was 7.23 (+/-2.11) months with a minimum of 1 month after the first injection. Topical anti-glaucomatous topical medication was initiated if ocular hypertension occurred (i.e. if a pressure reading above 21mmHg was measured).

The normally distributed data was analyzed using an unpaired t-test. For interindividual comparisons only one randomly selected eye per patient was taken for statistical analysis. For intraindividual comparison, the four patients with both eyes treated were included in the analysis.

## RESULTS

**Fig. (1):**
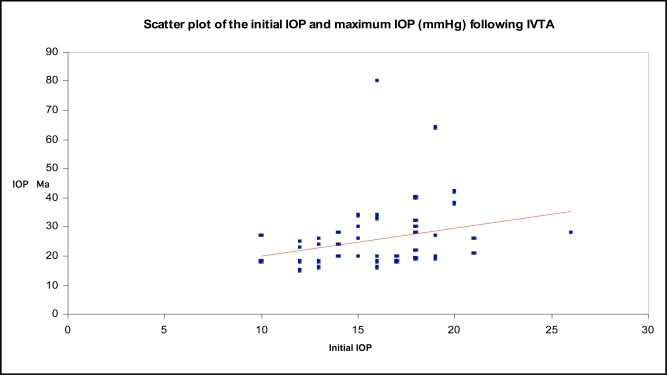
Scatter plot of initial IOP versus maximum IOP following IVTA.

Intraocular pressure increased significantly (p<0.001) from 16.08 (3.284) mm Hg (range 10-26 mm Hg) to a mean maximum of 26.1 (11.79) mm Hg (range 15-80 mm Hg) postoperatively (Fig. **1**). The differences between the IOP measurements before the injection and the postoperative examinations were significant (p<0.05) for the examinations performed 1 week or later after the injection.

A rise in IOP to values higher than 21 mm Hg was observed in 28 (53.8%) eyes. The elevation usually occurred after 7.5 weeks (+/-7.07).

One of the two patients with a current diagnosis of POAG demonstrated an IOP rise warranting treatment, the other needed no additional treatment.

The post-injection rise of IOP to values higher than 21 mm Hg was statistically independent of sex (p=0.37). The presence of diabetes mellitus however, demonstrated a marked influence on the rate of a postoperative elevation of IOP (p=0.05).

**Fig. (2):**
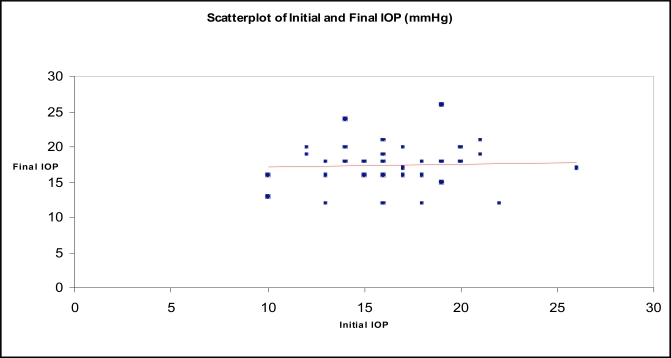
Scatter plot of the initial and final IOP.

Within the group of patients with a postoperative rise of IOP, in all but one eye, IOP could be lowered to normal levels with topical medication alone without developing glaucomatous optic nerve head changes (Fig. **2**).

One eye developed an elevation of IOP to 80mmHg following IVTA. An anterior chamber paracentesis was promptly performed resulting in an effective reduction in pressure to 21 and then to a final IOP of 12.

All eyes which developed a secondary ocular hypertension after a second intravitreal injection of triamcinolone acetonide had also shown a rise in IOP after the first intravitreal injection. In the absence of a pressure rise after the first injection, no eyes showed an increase in IOP after a second injection was performed.

Eight (80%) of the ten patients who received an intravitreal injection of triamcinolone acetonide into both eyes with a time interval of 3.5 to 6 months between the injections, developed ocular hypertension in both eyes at about the same time after the injection (mean 6.7 weeks).

All five patients (11.9%) in the study with a positive family history of glaucoma developed an IOP rise above 21 mmHg. The IOP-time relationship is shown in Table **1**.

## DISCUSSION

The findings of this British study from a predominantly Caucasian population in the South-East of England concur with previous studies from Europe and USA [4-8].

One of the major side effects of intravitreal injection of triamcinolone is a steroid induced elevation of IOP [5,9,10]. The mechanism by which this happens is not completely understood. Immediately after the injection, there is said to be an expansion of eye volume and a corresponding increase in the rigidity of eye, as demonstrated by the Friendenwald Equation [11]. 1 week to 2 months following injection in 40% of patients, the increased resistance is thought to be due to a change in morphological and mechanical changes in the trabecular meshwork and most IOP changes return to baseline within 3-5 months. In 1% of patients, IOP fails to return to baseline, and although filtration surgery is popular, some treat the cause rather than effect and advocate vitrectomy for uncontrolled IOP [12].

The result of the present study showed that a rise in IOP to values higher than 21 mm Hg can be expected to occur in 53.8% of eyes. This is comparable to results found from other large studies which looked at the incidence of IOP rise with IVTA [4,7].

A predictive factor for the rise in IOP may have been the presence of glaucoma before the injection. However, there were not enough cases with underlying POAG in the cohort to study the statistical significance of this properly*.* Other parameters such as gender did not show a marked influence on frequency and amount of elevation of IOP. The presence of diabetes mellitus however, demonstrated a marked influence on the rate of a postoperative elevation of IOP (p<0.05).

From a clinical standpoint it is important that in all but one eye, the IOP could be controlled by topical antiglaucoma treatment, or returned to normal values after the intravitreal steroid crystals had resolved about 6 months after the injection. Furthermore, it is important to note that a rise in IOP after a second intravitreal injection did not occur if there was not one found after the first.

Unexpectedly, three of the patients who received intravitreal steroid injections in both eyes had an IOP rise above 21mm Hg in one eye only. The reason for this is not clear.

The dose of steroid used in the present investigation was the same as other clinical studies reporting on the intravitreal injection of triamcinolone acetonide [1, 2, 11, 13, 14-19]. As expected, the incidence of IOP rise was found to be similar to these studies. Jonas *et al.* [10] found the incidence of IOP rise to be 50% following a dose (25 mg) of triamcinolone acetonide and the IOP was controlled following treatment without the development of major damage to the optic disc. This finding is comparable to other studies which used a lower of dose of 4mg of IVTA. Although, there is thought to be a linear dose-dependent IOP response immediately following an injection, as demonstrated by the Friendenwald equation [11] the precise mathematical relationship between steroid dose and subsequent IOP response over time is yet to be determined.

The aetiology of steroid induced glaucoma is not entirely understood. There is some evidence to suggest that the accumulation of glycosaminoglycans in the extracellular matrix (ECM) of the trabecular meshwork or increased production of trabecular meshwork induced glucocorticoid response (TIGR) protein may be responsible for this. However, in a study by Kee at al there was found to be no relation between the TIGR gene and steroid induced glaucoma [6]. None of the patients in the study with steroid induced glaucoma were found to have the TIGR gene mutation. Furthermore, a study by Fingeret *et al. *showed that variations in the MYOC gene (the animal version of the TIGR gene) did not seem to be responsible for the development of steroid induced glaucoma [20]. The ECM changes are very likely under control of transforming growth factor-beta2 (TGF-beta2), which is found at high concentrations in the aqueous humor of patients with primary open-angle glaucoma [21, 22]. Additional factors are thrombospondin-1, which activates TGF-beta2 *in vivo*, and connective tissue growth factor, which is an important downstream mediator of the effects of TGF-beta2 on trabecular meshwork ECM turnover. In contrast, bone morphogenetic protein-7 (BMP-7) strongly antagonizes fibrogenic actions of TGF-beta2 on human trabecular meshwork cells, indicating that a pharmacological modulation of BMP-7 signaling might be a promising strategy to treat primary open-angle glaucoma [21]. The effect of TGFbeta2 and glucocorticoids on cultured trabecular meshwork cells show typical changes in formation of ECM components and of stress proteins. Dexamethasone and oxidative damage also lead to increase of trabecular meshwork inducible glucocorticoid response (TIGR) protein [23].

In the present study, all five patients (11.9%) with a positive family history of glaucoma developed an IOP rise above 21 mmHg. This suggests that steroid-induced glaucoma may be hereditary or a family history of glaucoma may predispose someone to higher IOP rise following IVTA.

In conclusion, the data from this study suggest that the intravitreal injection of triamcinolone acetonide at a dosage of 4mg leads to a secondary ocular hypertension in about 54% of the eyes treated. The rise in IOP is reversible at 6 months after the injection and can almost always be controlled by topical hypotensive medication alone. A rise in IOP following IVTA is common and poses a strong argument in favour of treating all patients with ocular hypotensive medication both pre and post operatively. It also underlies the importance of informing patients about this risk when consenting pre-operatively. A family history of glaucoma should be elicited in all patients, as this may be a predisposition to higher IOP rise following IVTA.

Further research is required to scientifically establish the aetiology of steroid-induced glaucoma and target possible areas of prevention and treatment.

## Figures and Tables

**Table 1:** The Relationship between Mean IOP Rise and Time Following IVTA

	Mean IOP mmHg
Baseline	16.08 (3.3)
1 month	18.4 (7.1) p<0.05
3 months	18.2 (7.3) p<0.05
6 months	16.9 (4.4)
